# Combined Longitudinal and Surface Acoustic Wave Analysis for Determining Small Filling Levels in Curved Steel Containers

**DOI:** 10.3390/s22093476

**Published:** 2022-05-03

**Authors:** Michael Metzenmacher, Alexander Beugholt, Dominik Geier, Thomas Becker

**Affiliations:** Institute of Brewing and Beverage Technology, Technical University of Munich, 85354 Freising, Germany; michael.metzenmacher@tum.de (M.M.); alexander.beugholt@tum.de (A.B.); tb@tum.de (T.B.)

**Keywords:** longitudinal wave, surface acoustic wave, ultrasound, ultrasonic, small filling level

## Abstract

Measurement of the small filling levels in closed steel container systems is still a challenge. Ultrasound, however, is a sensitive and non-invasive technique and is suitable for online monitoring. This study describes a new ultrasonic sensor system for sensing small filling levels using longitudinal and surface acoustic wave analysis. The sensor system consists of one transducer for the longitudinal wave analysis and two transducers for the longitudinal and surface acoustic wave analysis. All transducers were mounted to the outer wall of the steel container, ensuring non-invasiveness, and a filling level ranging from 0 to 5 cm was investigated. Combining both approaches, a consistent determination of small filling levels was achieved for the entire measuring range (R^2^ = 0.99).

## 1. Introduction

Online analysis is the foundation for the automation and digitisation of manufacturing processes [1,2,3]. The determination of fluidic filling levels is critical for various industrial processes, including automation and ensuring product and process quality [4] Several measuring concepts are used for this purpose, including mechanical measurements (float, differential pressure, etc.), conductivity measurements, capacitive measurements, optical methods, microwaves, radar and ultrasonic measurements [5,6,7]. Nevertheless, invasive measuring principles, such as floats, conductivity and capacitive measurements, are unsuitable due to sensitive safety issues and contamination risks. The optical, capacitive and conductive properties of the substances to be measured limit the operational capability of the utilised measurement method, too. Acquisition costs also play a major role, making cost-intensive technologies, such as microwaves and radar, less likely to be used for most applications. However, due to their limitations, the previously mentioned measurement methods have major food and life science deficiencies. In contrast, ultrasonic-based methods exhibit the requisite properties, such as non-invasiveness, applicability to various media and cost-effectiveness [8]. The use of ultrasonic sensors has recently significantly increased in various areas of process monitoring. In comparison to many other techniques, ultrasound provides a quick, cost-effective and non-destructive tool for analysing process and product parameters [9,10,11]. Ultrasound is appropriate for various applications due to its non-invasiveness, particularly for the product-sensitive process steps in life sciences [12,13,14,15]. The generation of ultrasound waves is mostly realised by piezoceramic transducers, which convert electrical energy into mechanical energy and vice versa. Ultrasonic transducers can be used for a wide temperature range (up to 300 °C) and offer long-term stability and reliability for industrial applications [16]. Depending on the application’s requirements, specific piezoceramics can be used to design the transducers [17]. Ultrasound is described as mechanical waves with higher frequencies than those audible to humans (>20 kHz). Depending on the medium, acoustic waves propagate in different types of waves, which can be divided into longitudinal, transverse and surface waves [18,19,20]:

- Longitudinal waves (LW): With LW, the direction of oscillation of the individual particles is identical to the sound wave’s propagation direction. During wave propagation, compression and decompression areas are formed. LW propagate in gases, liquids and solids.
- Transverse waves (TW): In TW, the particle motion is perpendicular to the wave propagation. In contrast to LW, the speed of sound is significantly reduced. TW can only form in media with shear modulus, mainly solids.
- Surface acoustic waves (SAW): SAW consist of a combination of longitudinal and TW and propagate particularly at the interfaces between the phases of solid/liquid and solid/gaseous. The sound velocity of SAW is comparable to that of TW.

**Figure 1:**
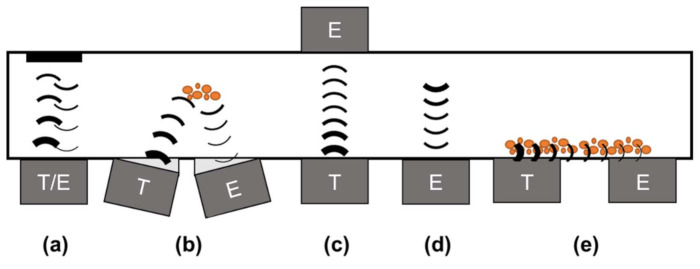
Ultrasound-based measuring setups with transmitter (T) and emitter (E): (**a**) reflection measurement in pulse-echo mode, (**b**) reflection measurement in pitch-catch mode, (**c**) transmission mode, (**d**) emission mode, (**e**) surface acoustic wave measurement in pitch-catch mode.

Furthermore, there are three basic ultrasound measurement methods: reflection, transmission and emission (see Figure 1). Reflection measurements (see Figure 1a,b) can be realised with one (pulse-echo mode), two or more transducers (pitch-catch mode) on the same side. Here, the transmitter couples the acoustic wave into the medium, while the emitter records the wave reflections at boundary surfaces. Therefore, the acoustic wave always passes through the medium twice. This method allows for determination of the time of flight (TOF) of the acoustic wave and other acoustic parameters, such as the attenuation (amplitude), the frequency shift of sensor-specific acoustic waves and impedance (*Z*). The acoustic impedance *Z* can be described with Equation (1):

(1) $$Z=\frac{p}{u}=\rho c$$

where *p* is the sound pressure, *u* is the ultrasound velocity, 𝜌 is the density, c is the acoustic velocity and *Z* is the impedance.

Reflections occur at the interfaces between two media with different impedances. The reflection coefficient *R* is the ratio of detected sound energy *p_d_* (echo energy) to emitted sound energy *p_e_*, from which conclusions regarding the qualities of the analysed media can be formed. The reflection coefficient *R* can be described with Equation (2):

(2) $$R=\frac{p_{d}}{p_{e}}=\frac{Z_{2}-Z_{1}}{Z_{2}+Z_{1}}$$

where *Z*_1_ is the acoustic impedance of medium 1, and *Z*_2_ is the acoustic impedance of medium 2. 

The acoustic signal is emitted by a transducer (transmitter) and recorded by another transducer (emitter) for transmission measurements (see Figure 1c). The acoustic wave only penetrates the medium once. The sound propagation time (TOF), attenuation (amplitude) and frequency shift are all determined using this method. Media changes can be rectified on the basis of attenuation of the reflected signal at different interphases. Surface waves (see Figure 1e) can also be recorded and analysed in this way. For emission measurements (see Figure 1d), sound signals are recorded and emitted by the process itself. Changes in the process influence the acoustic signals in both time and frequency domains. These signal alterations characterise the process and can be determined via ultrasonic sensors.

However, the various measurement methods usually use similar sound parameters (see Table 1). Table 1 illustrates the challenges of acoustic measurements. Measurements in closed systems, such as curved steel containers, combine signals of all measuring methods due to their geometry. While only a few sound parameters are determined, many applications can be characterised by alterations of these sound parameters. Ultrasound sensors can be removed at the top, bottom or side of a container using various measuring methods [21]. Air-ultrasound can be used to determine the filling levels of solid and liquid media from the top. Acoustic waves are transmitted via the air and reflected at the air/medium interface. The use of appropriate sound frequencies allows for exact assessments of large and small filling levels [22]. Nevertheless, this type of measurement is invasive. Ultrasonic sensors can also be used as limit switches. The impedance difference between steel, water and air can be used to calculate the filling level by attaching several sensors to the container’s side [23,24,25,26]. To obtain exact filling levels with this method, the sensor must be moved manually and is therefore only suitable for manual checks. When an ultrasonic transducer is attached to the bottom side of the container, the ultrasound wave is not travelling through the air, but through the container wall into the liquid phase and reflected at the phase boundary (liquid/air). This results in additional reflections in the container wall because of the high impedance difference between steel and water/air, which strongly affects the measurement of the small filling levels during pulse-echo mode.

Due to the near field region’s effects on ultrasonic waves through thin container walls (<5 mm), it is even more challenging to generate stable signals [23]. However, large filling levels in containers be achieved with a single transducer due to temporal isolation of the ultrasound signals [27,28]. Ultrasonic sensor systems in the industry and research have predominantly used TOF analysis for level measurement. A new approach featuring the combination of longitudinal, transverse and surface waves for level measurement was presented by Raja et al. [29,30]. The acoustic signal was coupled into the medium via a waveguide, realised with an invasive steel bar and recorded in pulse-echo mode. Due to differences in the propagation times of the individual wave types, each wave type could be analysed individually in the time and frequency domains. However, this method is invasive and therefore not suitable for processes with sensitive media. Nonetheless, it shows potential for combining different wave types for analysis. Measurement of the filling level in a steel vessel using guided waves within the wall of a steel vessel was introduced by Gao et al. [31]. After simulation of the guided waves, they showed a correlation of the TOF between the transducers and the filling level. Nevertheless, this method only includes the flight time and therefore shows an error of ±3.7 mm for a measuring range of 0–100 mm. Furthermore, the measurements were conducted on a planar steel plate and an almost ideal coupling of the ultrasonic sensors.

This study presents a novel method for combining longitudinal and surface acoustic wave analysis to monitor small filling levels in curved container systems non-invasively. Curved containers complicate both the coupling of the sensors and the measurement of small levels due to increased scattering and focussing effects. These difficulties can be overcome by combining the two methods. Ultrasonic transducers are attached to the outside of the container wall to avoid influencing the process medium. The so-called acoustic features are extracted from the ultrasonic signals and correlated with the filling level through chemometric analysis.

## 2. Materials and Methods

### 2.1. Experimental Setup

The ultrasonic transducers were excited by a rectangular 100 V excitation of 450 ns with three pulses in a row to obtain stable signals. A custom-designed measurement apparatus was used in the experimental setup for filling level measurement. The signals were recorded with 16-bit vertical resolution and 50 MHz time resolution. The self-made transducers consist of a piezoceramic disk (piezoelectric material: lead meta-niobate (Noliac Ceramics s.r.o.), 30 mm diameter, the main frequency of 1 MHz) encapsulated in resin and tungsten-based backing material to avoid ringing. Both the housing material and the piezoceramic disk are cylindrical shapes. The cylindrical container is composed of steel (P245NB, radius 14.7 cm and width 11.6 cm) with side parts consisting of PMMA to enable visual control of filling levels. The volume of the container system can be described using Equation (3).

(3) $$V=lr^{2}\arccos\left(\frac{r-h}{r}\right)-l\left(r-h\right)\sqrt{r^{2}-{\left(r-h\right)}^{2}}$$

where *V* is thevolume, *l* is the width, *r* is the radius and *h* is the filling level. 

**Figure 2:**
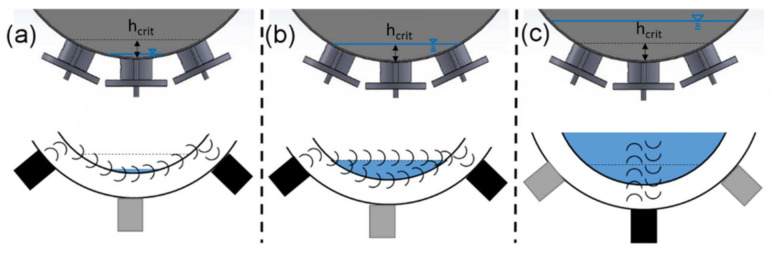
Schematic ultrasound sensor setup. Active transducers are highlighted in black. (**a**) Filling level below the critical level (hcrit): transmission measurement with SAW detection in pitch-catch mode only (black transducers). (**b**) Filling level at the critical level (hcrit): transmission measurement with SAW (steel–water surface) and with longitudinal wave (water) detection in pitch-catch mode. (**c**) Filling level above the critical level: reflection measurement with longitudinal waves for TOF determination in pulse-echo mode.

Three transducers were attached to the outer surface of the steel container using acoustic coupling grease (X380030100, KROHNE Messtechnik GmbH) and equal downforce (see Figure 2). The sound energy of the ultrasonic sensors enters the container at an angle, which also creates surface acoustic waves due to the curved surface. This leads to the fact that hardly any LW are formed and therefore cannot be detected. For a critical filling level (at the height of the angled sensors), shown in Figure 2b, SAW at the steel–water surface and LW through water can be detected. For small filling levels below the critical level, shown in Figure 2a, only SAW can be detected due to the high reflection factor of acoustic waves between steel and air. A filling level greater than the critical filling level does not affect the SAW measurement any more but can be measured with the third transducer via TOF (see Figure 2c). The positioning of the three sensors, therefore, results from the minimum filling level, which can still be measured with the lower sensor using TOF analysis. The outer sensor pair was therefore set to a height of 1.2 cm (resulting angle: 30°).

### 2.2. Experimental Work

The acoustic measurements of the filling level in the container system were performed for different filling levels. Increments of 25 mL increased the filling level for each measurement. The container was filled by water, which was measured in a volumetric flask before to filling. The resulting filling level was determined using Equation (3). For each filling level, 30 ultrasonic signals were recorded. A total of five replication measurements were realised throughout the measuring range. At first, the filling level was determined by only one ultrasonic sensor attached to the bottom apex of the steel container. Measurements were taken from 0 to 5 cm. Therefore, the filling level was determined by the TOF measurement method only. For filling levels smaller than 1.2 cm (h_crit_), the interpretation of the TOF measurement was impossible due to reflection issues. The critical filling level (1.2 cm) depended on different factors (wave length, steel wall thickness, wall material, etc.) and was determined experimentally. To obtain accurate measurements below these limits, the other transducers were attached at this height at the outer part of the steel container resulting in an angle of 30°. In further measurements, the filling level was determined via SAW using the two transducers attached with an angle at the container. Measurements were taken from 0 to 5 cm too. The two methods were combined in a final step, and a model was created to ensure exact measurement of the whole range of small filling levels in a steel container.

### 2.3. Ultrasound Signal Processing

Only the TOF was analysed for reflection measurements with one transducer (pulse-echo mode). The filling level can be determined using the measured TOF, at the known ultrasound velocity, considering the fluid in the tank (water: 1484 m/s at 20 °C). The ultrasound signals were filtered with an adapted eighth order Butterworth filter (0.85–1.15 MHz). The signal was also processed to ensure a stable analysis, creating an envelope curve for the ultrasound signals (McVittie & Atlas, 2008). The signal, processed in this way, was used for TOF prediction to evaluate the first echo in the ultrasound signal. The exact ultrasound velocity, however, depends on many factors (temperature, pressure, etc.). Therefore, a correction factor was introduced to consider this uncertainty. The correction factor was determined by reference measurements (F = 1.005, data not shown). The thickness of the steel wall must also be taken into account. The calculated filling level is therefore derived using Equation (4):

(4) $$V=lr^{2}\arccos\left(\frac{r-h}{r}\right)-l\left(r-h\right)\sqrt{r^{2}-{\left(r-h\right)}^{2}}$$

where *h* is the filling level, *c* is the ultrasound velocity, TOF is the time of flight, F is the correction factor and *d* is the thickness of the steel wall. 

**Figure 3:**
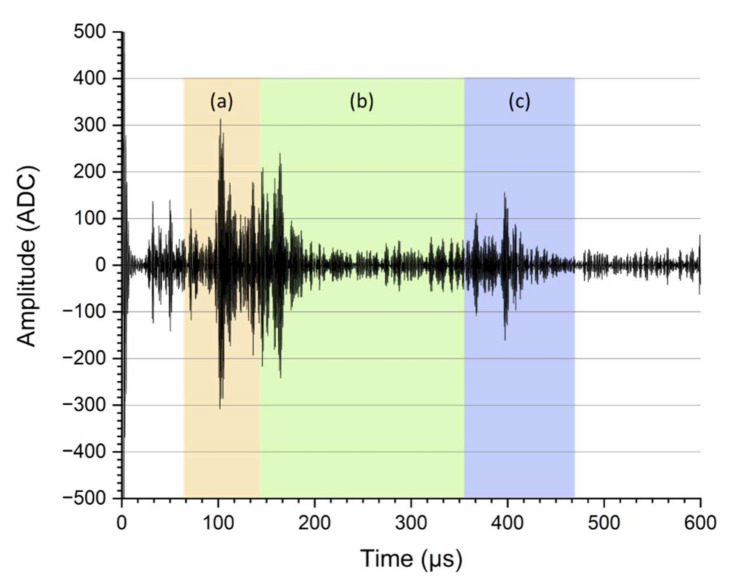
SAW ultrasound signals in transmission mode are divided into three parts for subsequent analysis: (**a**) SAW detection in pitch-catch mode, (**b**) echo detection of SAW, (**c**) longitudinal wave detection in pitch-catch mode.

For transmission measurements with two transducers, the SAW and LW were analysed. Due to the curvature of the container, superpositions occur, which influence the ultrasonic signals. Therefore, physical dependencies of the features can only be explained indirectly using a data-driven approach. To create a valid model, 70% of the measured ultrasonic signals (about 2500 ultrasonic signals) were used for modelling and 30% for validation. A Gaussian model from the previously acquired and selected acoustic features (N = 10) was used for prediction. To create the features, the signal is analysed in the time domain and the frequency domain using a fast Fourier transformation (FFT). To remove part of the noise of the signals in the time domain, an adapted eighth order Butterworth filter for the respective frequencies was used (0.85–1.15 MHz). The signals obtained were taken for the subsequent feature analysis for the time domain. The ultrasonic signals processed for the time domain were divided into three parts representing (a) the initial SAW detection in pitch-catch mode, (b) the echo detection of SAW and (c) the longitudinal wave detection in pitch-catch mode (see Figure 3). Based on the physical effects of attenuation and TOF related to the ultrasound wave propagation, the features were created for each signal part. Therefore, three features (maximum amplitude, time location of maximum amplitude and sum of amplitudes in the selected part) of each signal part (a, b and c) were computed. These time-domain features can be described with Equations (5)–(7).

(5) $$maximum\;amplitude=\underset{x}{max}\left(Ax\right),\;x\;\in \left\{x_{n},r_{1}\leq n\leq r_{2}\right\}$$

(6) $$time\;location\;of\;maximum\;amplitude=arg\;\underset{x}{max}\left(Ax\right),\;x\;\in \left\{x_{n},r_{1}\leq n\leq r_{2}\right\}$$

(7) $$sum\;of\;amplitudes=\overset{r_{2}}{\underset{n=r_{1}}{\sum }}\left|x\left(n\right)\right|$$

where *A* = signal value, *x* = time, *n* = discrete sampling point, *x_n_* = discrete time, *r_1_* = lower time boundary and *r_2_* = upper time boundary. 

**Figure 4:**
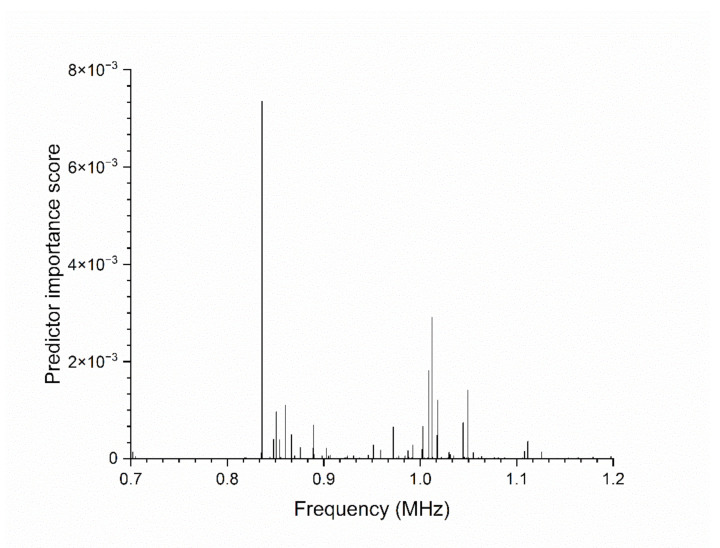
Predictor importance score of the frequencies for feature selection around the main frequency of the ultrasound sensor (1 MHz).

To improve comparability, each feature was normalised to the signal energy of the entire ultrasonic signal. Subsequently, the predictor importance score of all time-domain features (the three features for the three signal parts (a, b, c), N_time_ = 9) was predicted using a least-squares boosting trees (LSboost) analysis [32]. To prevent the model from overfitting, only the five most important time-domain features were selected for further analysis. Similarly, the frequency shift was determined in the frequency domain as a result of the data-driven evaluation for every signal part (a, b, c, see Figure 3). Due to the main frequency of 1 MHz of the ultrasonic sensor, the predictor importance (LSboost) was calculated only for the frequency band (gained via FFT) around the main frequency (0.7–1.2 MHz), including and extending the frequency range of the bandpass filter set up by a small amount (see Figure 4).

Only the five most important frequency features (wavelengths with highest predictor importance scores) were selected (total number of frequency domain features, N_frequency_ = 500) for further analysis to allow a balance between frequency and time-domain features and to avoid overfitting. For predicting the filling level, the ten features (five time domain, five frequency domain) were used for a Gaussian process regression [33]. The Gaussian process regression is, therefore, a nonparametric kernel-based probabilistic model. Using a rational quadratic Gaussian process regression (rational GPR) allows for modelling of data with multiple varying scales. As ultrasonic signals in the near field of the transducers are noisy from experience, the systematic relation of the features to the filling level cannot be observed directly. Therefore, an additional parameter to describe normal noise is integrated into the model. The rational GPR model can be described with Equation (8).

(8) $$k\left(x_{i},x_{j}|\theta \right)=\sigma_{f}^{2}\left(1+\frac{r^{2}}{2\alpha \sigma_{i}^{2}}\right)$$

where

(9) $$r=\sqrt{{\left(x_{i}-x_{j}\right)}^{T}\left(x_{i}-x_{j}\right)}$$

where *θ* is the maximum of posteriori estimates, *σ_f_* is the signal standard deviation and *α* is the non-negative parameter of the covariance. 

**Figure 5:**
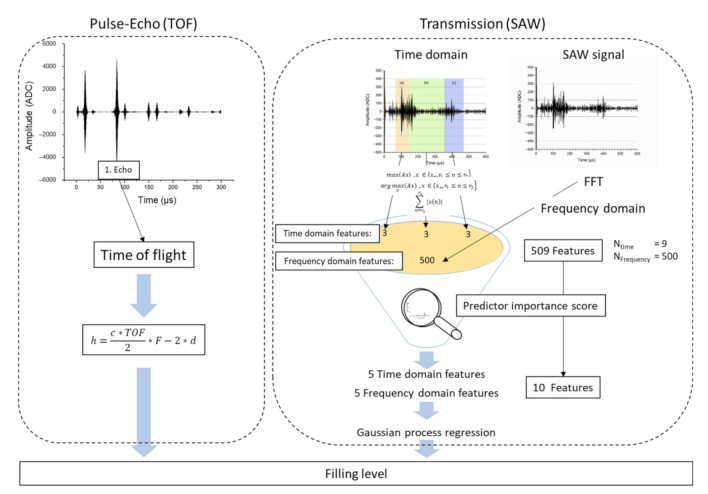
Signal processing strategy for model based pulse-echo (TOF) analysis (**left**) and transmission (SAW) analysis (**right**) using a data driven model. Both methods are calculated seperately and estimate the filling level.

To select and train the GPR model 70% of the measured data where used. For testing, 30% of the data were used. Therefore, two measurements were carried out for each measurement cycle: pulse-echo measurement (see Figure 2c) using the TOF model and transmission (SAW) measurement (see Figure 2a,b) using the rational GPR model. The whole ultrasonic signal processing strategy concerning pulse-echo measurement (TOF) and transmission (SAW) measurement for filling level detection is outlined in Figure 5.

## 3. Results and Discussion

### 3.1. TOF Measurement

**Figure 6:**
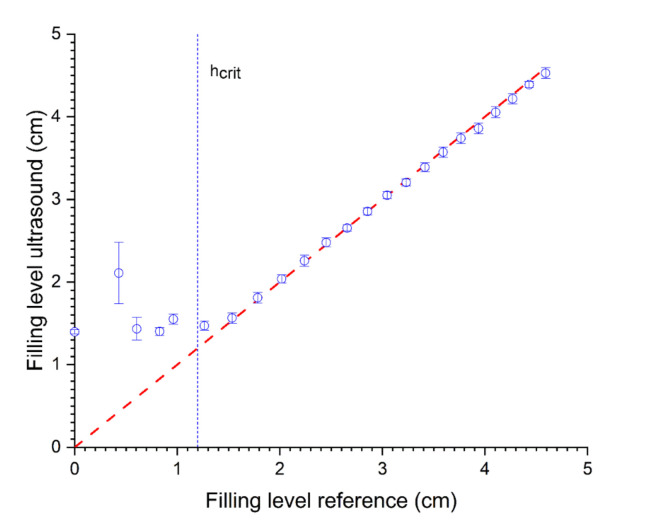
Relationship between predicted levels with TOF using one transducer in reflection mode and reference (volumetric determination). R^2^ = 0.81 and RMSE = 0.63 (N = 5).

To experimentally examine the change in TOF and the method’s accuracy, the ultrasonic signals (N = 5 per filling level) were taken for different filling levels. The filling level was calculated using Equation (4). For prediction of the filling level for levels larger than the h_crit_ (>1.2 cm), TOF measurements show a good approximation, whereas when using only one transducer, the smaller levels cannot be determined correctly (RMSE = 1.02 and R^2^ = −11.57 for <h_crit_, RMSE = 0.06 and R^2^ = 0.99 for >h_crit_, see Figure 6 and Table 2). Even the standard deviation of the ultrasonic measurements increases with a lower filling level.

Due to multiple reflections in the steel wall of the container, the real water level echoes cannot be detected accurately using only pulse-echo mode with one transducer mounted on the bottom apex of the container. Thereby, the initial echo signal is superimposed by echo signals resulting from the multiple reflections from the steel wall. The signal is damped by each reflection within the steel wall due to the reflection factor (see Equation (2)). Furthermore, the measurement is within the near field of the ultrasonic sensor with unstable signals [23]. This results in a characteristic time-dependent window where no filling level measurements are possible (dead time). Even machine learning approaches did not show sufficient results (data not shown). At the critical level, the echo signal of the filling level surface (liquid/air) can be separated from the steel reflection signals. Filling levels above this critical level, therefore, can be determined. This concludes that the TOF measurement is unsuitable for small filling levels and must be enhanced to obtain stable signals up to the critical filling level (1.2 cm).

### 3.2. SAW Measurement

**Figure 7:**
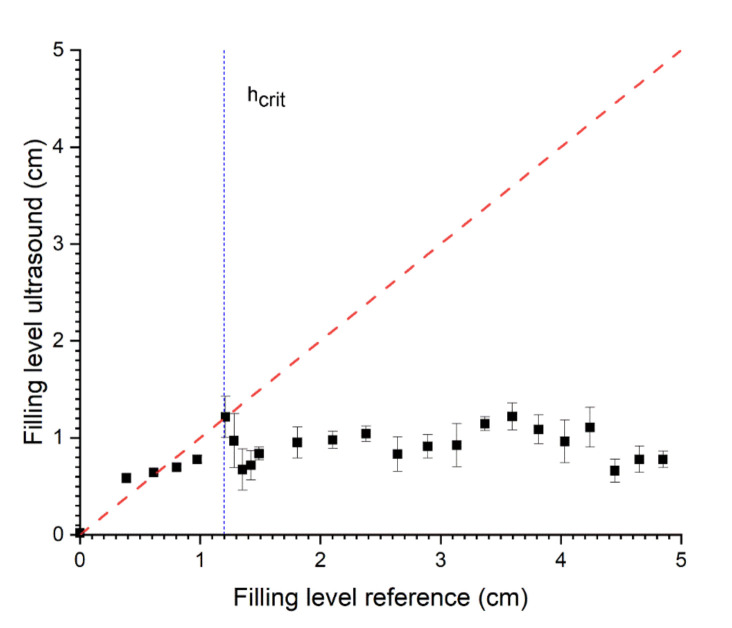
Relationship between the predicted level and SAW in pitch-catch mode with two transducers side-mounted and reference (volumetric determination). R^2^ = −1.02 and RMSE = 2.37 (N = 5).

The prediction of small filling levels using the SAW method between 0 and 1.2 cm can be predicted with an RMSE of 0.07, whereas for the whole range, an RMSE of 2.37 was determined (see Figure 7 and Table 2). Additionally, the R^2^ shows that the model is only suitable for filling levels <h_crit_ (R^2^ = 0.97, see Table 2) and does not apply for filling levels >h_crit_ (R^2^ = −3.81, see Table 2). In this series of filling level measurements, the standard deviation of the ultrasonic measurements increases for higher filling levels. Due to the arrangement of SAW ultrasound transducers, this detection method is limited to filling levels below the critical level (see Figure 1b). Above the critical filling level, there is hardly any change to the US-features because the US signal path is completely filled with water (see Figure 2b), and the boundary conditions (steel/water/air) are no longer changing. This leads to highly unstable signals and false measurements above the critical filling level (see Figure 7). Changing the angle of the sensor pair influences the limit of this measurement method. To optimise the measuring range of the combined method (TOF and SAW), the SAW method must be accurate up to the lower limit of the TOF method. Stable measurements regarding the TOF method using only one transducer can be obtained above 1.2 cm. Therefore, the angle of SAW sensors was adjusted to enable measurements within the range of 0–1.2 cm.

For small filling levels (<1.2 cm, critical level), the SAW method shows better results, whereas, for higher filling levels (>1.2 cm, critical level), the TOF method is more precise. 

### 3.3. Combined Measurement

**Figure 8:**
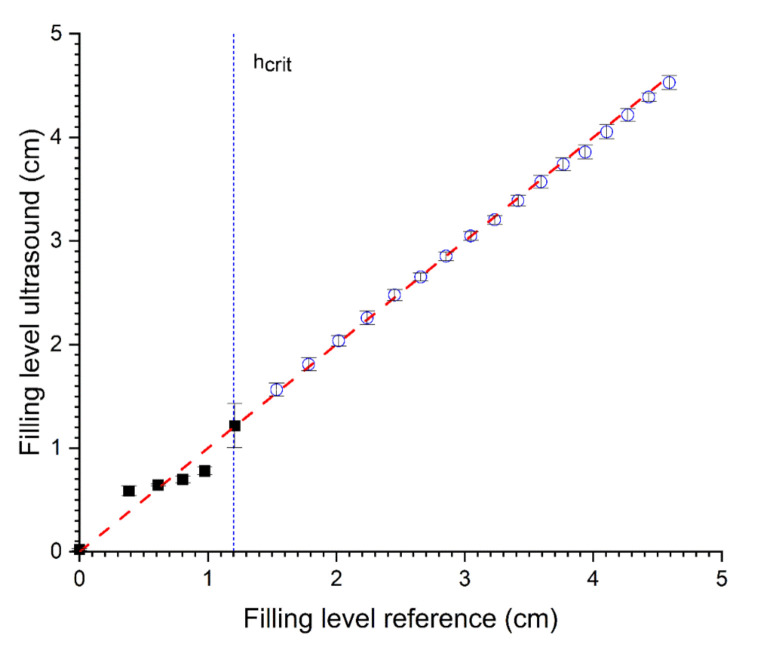
Relationship between the predicted level by combined prediction of filling level with SAW (0–1.2 cm) and pulse-echo prediction (>1.2 cm) and reference (volumetric determination). R^2^ = 0.99 and RMSE = 0.07 (N = 5).

Combining both methods (TOF and SAW) leads to a better prediction (RMSE = 0.07, R^2^ = 0.99, see Figure 8) of filling levels and can be used for small, as well as high, filling levels (up to 1 m, data not shown). By switching the methods at the critical level, the filling level can be predicted over the complete range. The switching point can be determined using the standard deviation of the ultrasonic measurements as the standard deviation differs greatly for both methods at certain filling levels. For lower filling levels, the standard deviation of the SAW method is smaller than for the TOF approach (see Table 2). Therefore, the SAW method is active and shows the right prediction, whereas, for the higher filling level, the standard deviation of the TOF method is smaller than the SAW method (see Table 2). Here, the TOF method is active and shows the right prediction. At the switching point (critical level), both methods gain slightly higher standard deviations, indicating the upper or lower limit of the specific measuring range; however, they show similar predictions. The combined method consolidates the advantages of both methods and covers the disadvantages at the same time (see Table 2). Therefore, it is possible to gain a precise ultrasound-based measurement system over the whole measuring range.

## 4. Conclusions and Future Scope

In this paper, a method combining the surface acoustic wave and TOF analysis to measure small filling levels is proposed. The developed ultrasonic sensor system was realised as a non-invasive clamp-on system and therefore does not affect the process. The established time of flight analysis using only one sensor in pulse-echo mode was extended with surface acoustic wave analysis methods using two sensors to enable measurements of small filling levels. However, the upper measurement range for the SAW is limited due to the provided angle between both sensors for round structures, whereas the frequency influences the spread and resolution as well as the lowest filling level measurement for TOF measurement. If the two measuring methods are well-coordinated, these disadvantages can be compensated.

Future research will extend the SAW approach with multiple sensors to improve accuracy and robustness for even smaller filling levels. In this study, the SAW model was adjusted to the entire measurement range. To enhance this model, a restriction to the filling levels <h_crit_ is beneficial. In addition, for the machine learning approach, the GPR model was selected as an example for proof of concept. By comparing different machine learning models (SVR, PLSR, neural network, etc.) and optimizing them, the approach shown in this study can be further improved. Another research direction would be to apply this combined method to other surface-dependent problems, whereas in former studies, only one sensor was used as shown, e.g., for fouling detection during spray drying [34,35], processing operation [36], characterisation of fouling layers [37] and monitoring of the cleaning process [38]. Especially concerning concepts of industry 4.0 and AI-based systems, combined acoustic-based sensors enable innovative approaches for industrial implementation.

## Figures and Tables

**Table 1:** Ultrasound-based measuring methods, utilised parameters and typical fields of application.

Measuring Method	Parameters	Fields of Application
**Reflection**	time of flight	distance, filling level, sound velocity, position, object structure, NDT (non-destructive testing), density, viscosity, concentration, movement, flow velocity
	frequency	
	amplitude	
	impedance	
**Transmission**	time of flight	concentration, particle size distribution in (low attenuation) emulsions and suspensions, sound velocity, flow velocity, density, viscosity, temperature
	frequency	
	amplitude	
**Emission**	frequency	process control
	amplitude	

**Table 2:** Summary of the R^2^ and RMSE of the models for the particular filling levels.

	R^2^			RMSE		
	<h_crit_	>h_crit_	Full Range	<h_crit_	>h_crit_	Full Range
**TOF**	−11.57	0.99	0.81	1.02	0.06	0.63
**SAW**	0.97	−3.81	−1.02	0.07	2.77	2.37
**Combined**	0.97	0.99	0.99	0.07	0.06	0.07

## Data Availability

The data that support the findings of this study are available from the corresponding author upon reasonable request.
